# Single Intramammary Infusion of Recombinant Bovine Interleukin-8 at Dry-Off Induces the Prolonged Secretion of Leukocyte Elastase, Inflammatory Lactoferrin-Derived Peptides, and Interleukin-8 in Dairy Cows

**DOI:** 10.1155/2012/172072

**Published:** 2012-08-07

**Authors:** Atsushi Watanabe, Jiro Hirota, Shinya Shimizu, Shigeki Inumaru, Kazuhiro Kimura

**Affiliations:** ^1^Dairy Hygiene Research Division, Hokkaido Research Station, National Institute of Animal Health (NIAH), National Agriculture and Food Research Organization (NARO), 4 Hitsujigaoka, Sapporo 062-0045, Japan; ^2^Intellectual Property and Technology Management Section, NIAH, NARO, 3-1-1 Kannnondai, Tsukuba 305-0856, Japan; ^3^Pathology and Pathophysiology Research Division, NIAH, NARO, 3-1-1 Kannnondai, Tsukuba 305-0856, Japan; ^4^Laboratory of Biochemistry, Department of Biomedical Sciences, Graduate School of Veterinary Medicine, Hokkaido University, Sapporo 060-0818, Japan

## Abstract

A single intramammary infusion of recombinant bovine interleukin-8 (IL-8) at 50 **μ**g/quarter/head, but not 10 **μ**g/quarter/head, induced clinical mastitis in three of four cows during the dry-off period, resulting in an elevated rectal temperature, redness and swelling of the mammary gland, extensive polymorphonuclear leukocyte (PMNL) infiltration, and milk clot formation from 1 to 28 days post infusion (PI). In the mammary secretions of the mastitic glands, high levels of IL-8 were sustained from 8 hours to 28 days PI, peaking at 1–3 days PI. The levels of leukocyte-derived elastase and inflammatory 22 and 23 kDa lactoferrin derived peptides (LDP) were also increased in the mammary secretions from the mastitic glands. In addition to the experimentally induced mastitis, the mammary secretions from the glands of cattle with spontaneous *Staphylococcus aureus* dry-period mastitis displayed milk clot formations and significant increases in their levels of PMNL counts, elastase, LDP, and IL-8, compared with those of the mammary secretions from the uninfected glands. These results suggest that after an intramammary infusion of IL-8 has elicited inflammatory responses, it induces the prolonged secretion of elastase, inflammatory LDP, and IL-8, and that long-lasting IL-8-induced inflammatory reactions are involved in the pathogenesis of *S. aureus* dry-period mastitis.

## 1. Introduction

Interleukin (IL)-8 is an inflammatory cytokine belonging to the CXC chemokine family that is produced by a wide range of cells, including lymphocytes, monocytes/macrophages, neutrophils, fibroblasts, and vascular endothelial and epithelial cells, in response to inflammatory stimuli, such as viral and bacterial infections. IL-8 plays pivotal roles in the recruitment and activation of polymorphonuclear leukocytes (PMNL), such as neutrophils [1, 2].

It has been reported that IL-8 is released in the body fluids and secretions of cows during the expulsion of the placenta [3] as well as those of cattle suffering from pneumonic pasteurellosis [4] or mastitis [5–11]. Accordingly, neutrophils, which are involved in placental expulsion [3] and bacterial clearance from the mammary gland [12, 13], are recruited and activated by IL-8 [14–16]. Moreover, the IL-8 gene expression level and polymorphisms in the IL-8 receptor-*α* (CXCR1) gene are related to the incidence and severity of diseases such as mastitis [17, 18].

Activated neutrophils release lactoferrin (Lf) [19] and proteases such as elastase [20, 21]. The latter produce inflammatory Lf-derived peptides (LDP) including 22 and 23 kDa peptides [22]. Inflammatory LDP containing the GQRDLLFKDSAL sequence, such as the 22 and 23 kDa peptides, induce IL-8 gene expression in bovine mammary epithelial cells [22], which are major sources of IL-8 production in the mastitic mammary gland [23, 24]. Therefore, it is suggested that a positive amplification loop for IL-8 production, involving the sequential release of Lf and proteases from activated neutrophils by IL-8, exists in cattle.

We have recently demonstrated that infusing recombinant bovine (rb) IL-8 into the teat cisterns of dairy cows during the drying-off period induces inflammatory reactions similar to mastitic symptoms including the infiltration of PMNL into mammary secretions, a decreased casein concentration, and the transient elevation of rectal temperature [25]. However, it is uncertain whether intramammary infusions of rbIL-8 induce the release of elastase, LDP, and newly produced IL-8 in mammary secretions. To clarify these points and obtain further information on the local response to intramammary infusion of rbIL-8, we monitored the release of these substances in a time-dependent manner after the administration of a single intramammary infusion of rbIL-8. In addition, we also examined their release in clinical dry-period mastitis caused by intramammary *Staphylococcus aureus* infection.

## 2. Materials and Methods

### 2.1. Recombinant Bovine IL-8

Recombinant bovine (rb) IL-8 produced in *Brevibacillus choshinensis *was secreted into the culture medium. The rbIL-8 in the medium was purified by passing it through two filtration membranes (cut-off molecular weight: 100,000 and 3,000, resp.) followed by SP-Toyo-pearl chromatography (Tosoh, Tokyo, Japan) [26]. The concentration of the rbIL-8 was determined by the Bradford method [27] using bovine *γ*-globulin as a standard. The purity of the rbIL-8 was over 90%, as judged by densitometric scanning of the gel after SDS-PAGE [26, 28]. The biological activity of the rbIL-8 was confirmed by a chemotactic assay with bovine neutrophils and was completely blocked with monoclonal antibovine IL-8 antibody [26].

### 2.2. Animal Welfare and Bacteriological Tests

The care and handling of all animals used in this study were approved by the Institutional Care and Use Committee for Laboratory Animals of the National Institute of Animal Health. The rectal temperature of the cows was checked twice a day, and the bacteriological content of their mammary glands was assessed by microbiological culture using sheep blood agar and mannitol salt agar plates [29]. The bacteria isolated from the culture were subsequently identified by 16S rDNA sequencing with using MicroSeq Identification Systems (Applied Biosystems, Foster City, CA, USA) [30]. The number of bacterial cells was examined by the pour plate culture method (nutrient agar).

### 2.3. IL-8-Induced Mastitis

Twelve clinically healthy Holstein cows (5 to 6 years of age) were used in the experiment on IL-8-induced mastitis, which was performed during lactation days 220–233. They were randomly named A–L and divided into three groups (4 cows in each). Cows A–D were administered an infusion of rbIL-8 (50 *μ*g in 5 mL of endotoxin-free sterile saline) into the left front teat cistern and an infusion of 5 mL of endotoxin-free sterile saline into the right front teat cistern immediately after the final milking, as described previously [25]. Cows E–H were given infusions of rbIL-8 (10 *μ*g) and saline into the left and right front teat cisterns, respectively. Cows I–L received no treatment and were used as normal drying-off control animals. Mammary secretions (10 mL each) were collected from the infusion sites on days -4 -1, and 0 (just before the challenge) and 8 hours, 1, 3, 7, 14, 21, and 28 days postinfusion (PI). All of the samples were subjected to bacteriological studies but no positive cultures were yielded, confirming that there were no unexpected infections.

### 2.4. Spontaneous *S. aureus* Mastitis during the Dry-Period

Quarter mammary secretions (10 mL each) were also obtained from 7 Holstein cows with naturally occurring clinical mastitis caused by intramammary *S. aureus* infection. All of the cows remained healthy, that is, without any mastitic symptoms or causative pathogens in any quarter, for at least one month before and one week after dry-off. Each cow suffered mastitis involving swelling and redness on the outer aspects of one mammary gland quarter from 2 days before the sampling (8–12 days after dry-off). The cows showed no significant systemic clinical signs except for a transient fever involving a temperature increase of 1–1.5°C at 1 or 2 days before the sampling and were not given any treatments before the sampling. The mammary secretions from the infected (*n* = 7) and uninfected (*n* = 21) quarters were collected 10–13 days after the start of the drying-off period and were subjected to bacteriological and biochemical analyses.

### 2.5. Polymorphonuclear Leukocyte Counts and the Observation and Treatment of Mammary Secretion Samples

After assessing the gross appearance of the mammary secretions, they were filtrated through two layers of surgical gauze to remove clots. The number of somatic cells in the filtrates was counted by direct microscopic examination [31], and then the number of PMNL was estimated [32]. Skimmed milk and whey samples were prepared [33] and stored at −80°C until use.

### 2.6. Quantitation of Bovine IL-8

The IL-8 concentrations of the mammary secretions were determined using the sandwich enzyme-linked immunosorbent assay (ELISA) in duplicate, as described previously [26].

### 2.7. Leukocyte Elastase Assay

The elastase activity in the whey sample was detected by zymography, in duplicate, according to the method described by Komine et al. [22], with human leukocyte elastase (Sigma-Aldrich, St. Louis, MO, USA) used as a standard.

### 2.8. Quantitation of Lf and LDP

The concentrations of Lf and LDP were determined by quantitative western blot analyses using bovine Lf (Sigma-Aldrich) as a standard, in duplicate, according to the previously reported method [22, 34]. Briefly, skimmed-milk samples were separated by SDS-PAGE (13.5% polyacrylamide gel) and then blotted onto a polyvinylidene difluoride membrane. After blocking the nonspecific protein binding sites of the membrane with Tris-HCl (pH 7.5) buffer containing 0.15% (w/v) NaCl and 2% (w/v) ovalbumin, the membranes were sequentially treated with rabbit antibovine Lf IgG (Life Laboratory, Yamagata, Japan) and horse radish peroxidase (HRP)-conjugated donkey anti-rabbit IgG. The bound HRP was visualized using a kit (ECL plus western blotting detection system) (GE Healthcare, Little Chalfont, Buckinghamshire, UK).

### 2.9. Statistical Analysis

Prior to the statistical analysis, the PMNL counts and bacterial colony-forming unit (cfu) data were logarithmically transformed to maintain a normal distribution. When milk clot formation was observed in the mammary secretions, no statistical analysis of the PMNL count data was possible since the milk clots involved large numbers of PMNL, which prevented accurate counting of the cells. To determine the differences between the samples from the cows with and without mastitic symptoms, or the effects of different treatments, the data were analyzed by two-way repeated measures analysis of variance and Tukey's multiple comparisons test. To determine the differences between the mammary glands that were unaffected and affected by naturally occurring dry-period mastitis, the data were analyzed using the Student's *t*-test. All data are presented as means ± SEM, and differences with *P* values of <0.05 were considered to be significant.

## 3. Results

### 3.1. Responses to IL-8-Induced Mastitis

Three of the four cows (A–C) given a single high dose (50 *μ*g/quarter/head) intramammary infusion of rbIL-8 at dry-off displayed rectal temperature increases of 1–1.5°C, which peaked at 32 or 48 hours PI, and redness and swelling of the mammary gland from 1 to 7 and 1 to 14 days PI, respectively, (data not shown). Cow D, which was given a high dose of rbIL-8, failed to show these symptoms. In addition, the cows given a low dose (10 *μ*g/quarter/head) single intramammary infusion of rbIL-8 (E–H) showed no clinical signs and no changes in the outer aspects of their mammary glands (data not shown). 

**Figure 1:**
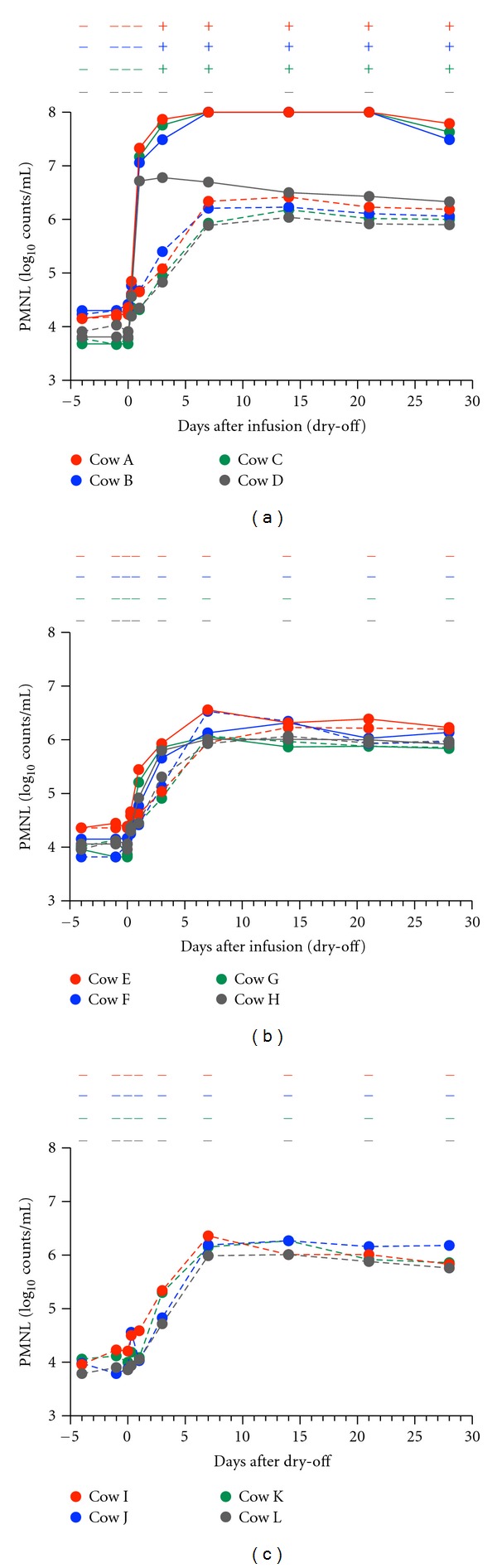
Polymorphonuclear leukocyte (PMNL) counts of the mammary secretions from rbIL-8-infused (solid line) and saline-infused (broken line) glands are shown in (a) and (b), respectively, and those of the mammary secretions obtained from the untreated glands during the drying-off period are shown in (c). Cows A–D and E–H were given 50 *μ*g and 10 *μ*g of rbIL-8, respectively. (+) or (−) in the upper part of each figure denotes the presence or absence of clots in the mammary secretions, respectively. As the PMNL counts were obtained after removing milk clots containing PMNL, the PMNL counts for the mammary secretions with milk clots were not accurate. PMNL counts of more than 10^8^ cells/mL were not counted.

The mammary secretions from the affected glands of the cows displaying clinical symptoms (A–C) contained large numbers of PMNL from 1 day PI and clots from 3 days PI, and the cell infiltration and clot formation lasted for 28 days (Figure 1(a)). The mammary secretions from the unaffected glands (A–D) displayed increases of up to 2.2 × 10^6^ cells/mL in the number of PMNL cells at 7 days PI without clot formation (Figure 1(a)). The mammary secretions from the unaffected cows (E–H) and from the cows that did not receive any treatment (I–L) also displayed increased numbers of PMNL cells, suggesting that this gradual increase in the number of PMNL can be attributed to mammary involution during the normal drying-off period (Figures 1(b) and 1(c)). The mammary secretions from the affected glands of the cows without clinical symptoms (D–H) tended to display initial increases in their PMNL counts, but the number of PMNL failed to exceed 10^7^ cells/mL thereafter (Figures 1(a) and 1(b)).

**Figure 2:**
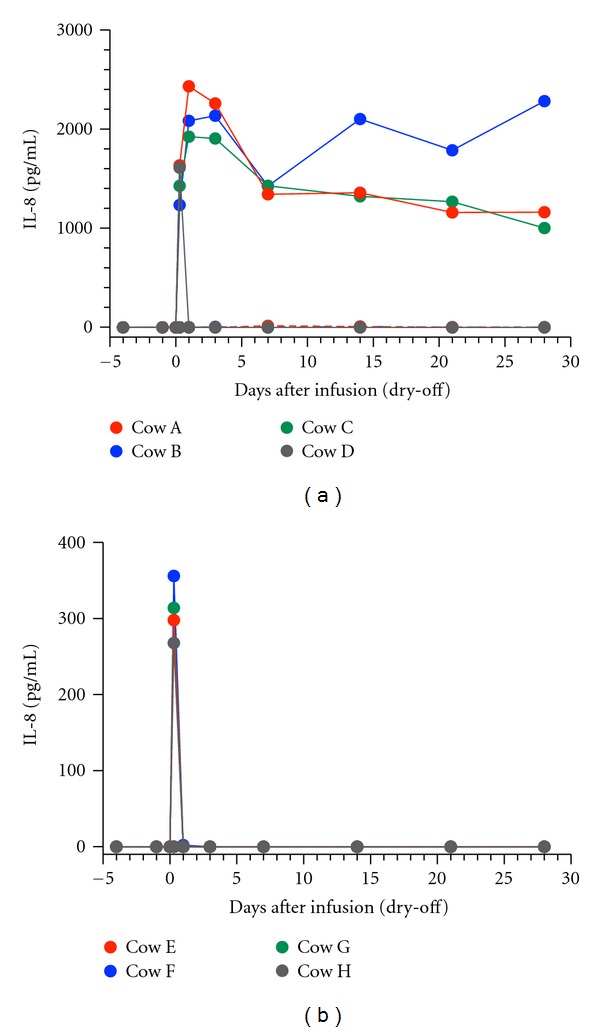
Changes in the IL-8 concentrations of the mammary secretions produced by the glands infused with 50 *μ*g (a) or 10 *μ*g (b) of rbIL-8 during the drying-off period.

The mammary secretions from the affected glands given high and low doses of rbIL-8 at 8 hours PI displayed IL-8 concentrations of 1480 ± 93 pg/mL and 309 ± 18 pg/mL, respectively. Moreover, the mammary secretions from the affected glands of the cows without clinical symptoms (D–H) did not contain IL-8 at 1 day PI or thereafter (Figures 2(a) and 2(b)). As the concentrations of IL-8 detected at 8 hours PI were roughly proportional to the dose of the rbIL-8 infusion administered and the IL-8 subsequently disappeared, it is likely that the IL-8 that transiently appeared in the secretions was derived from the infusions. In contrast, the mammary secretions from the affected glands of the cows with clinical symptoms (A–C) contained high levels of IL-8 at 1 day PI, which were sustained for 28 days PI (Figure 2(a)). These results suggest that IL-8 is continuously released during mammary gland inflammation.

**Figure 3:**
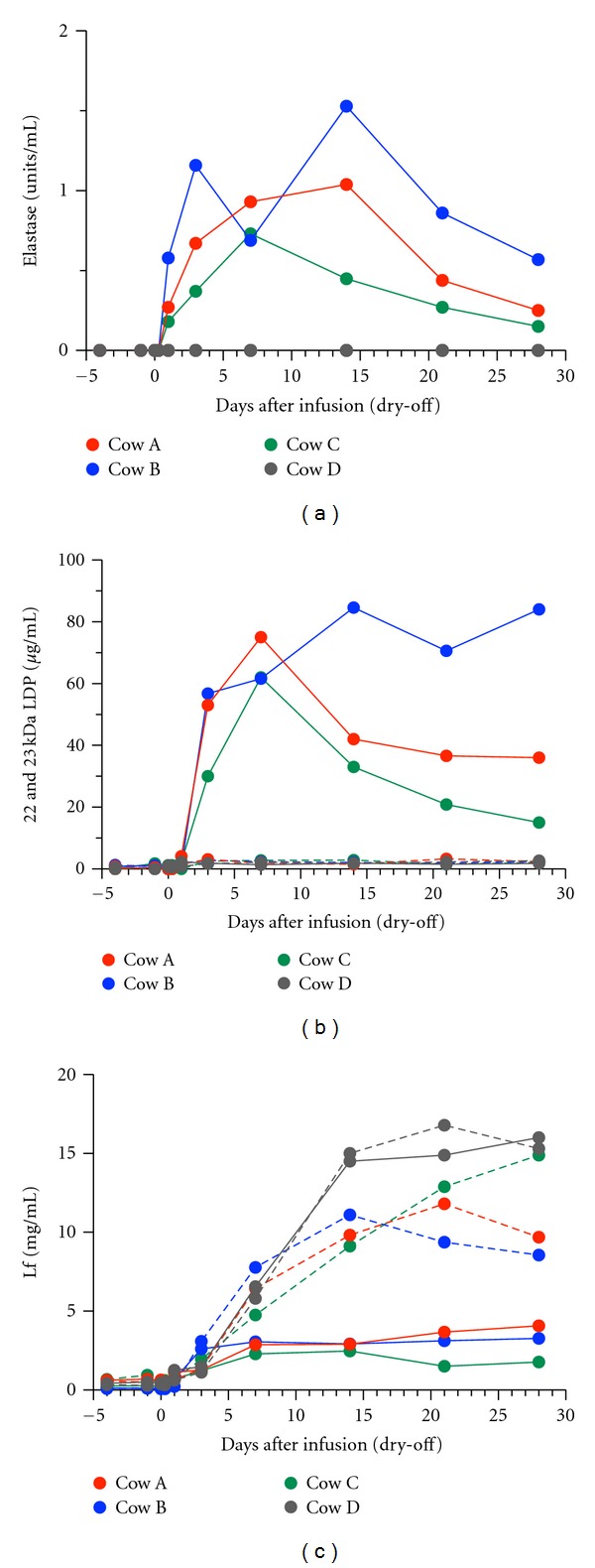
Changes in elastase activity (a) and the concentrations of 22 and 23 kDa LDP (b) and Lf (c) in mammary secretions from the glands infused with 50 *μ*g of rbIL-8 (solid line) or saline (broken line) at dry-off.

The mammary secretions from the affected glands of the cows with clinical symptoms (A–C) displayed high levels of elastase activity from 1 day PI, which lasted for 28 days PI (Figure 3(a)). The mammary secretions from the affected glands (A–C) contained 22 and 23 kDa inflammatory peptides (LDP), which were produced by the digestion of Lf by elastase, from 3 to 28 days PI, while the mammary secretions from the glands of one affected cow (D) and the unaffected glands of cows A–D did not (Figure 3(b)). In the latter mammary secretions, the concentration of Lf increased significantly from 3 to 28 days PI, as observed during normal dry-periods [35, 36]. However, in the former mammary secretions, the concentration of Lf increased marginally (Figure 3(c)) and was significantly lower than that in the latter secretions from 7 to 28 days PI, suggesting that the Lf was digested by proteases to produce LDP during this period. 

### 3.2. Responses to Spontaneous *S. aureus* Mastitis

Seven cows with clinical dry-period mastitis in a single quarter caused by intramammary *S. aureus* infection were recruited. In the mammary secretions from the uninfected quarters, a relatively low number of PMNL, low concentrations of 22 and 23 kDa LDP, and some Lf were found; however, no elastase or IL-8 was detected (Table 1). The mammary secretions from the quarters infected with *S. aureus* contained clots and higher numbers of PMNL. In addition, the mammary secretions contained high levels of elastase and IL-8 and significantly increased and decreased concentrations of LDP and Lf, respectively. 

## 4. Discussion

We have demonstrated that a single high dose (50 *μ*g/quarter/head) intramammary infusion of rbIL-8 induced the prolonged secretion of leukocyte elastase, inflammatory LDP, and IL-8 in dairy cows during the drying-off period. These phenomena were apparently related to the induction of mastitic responses by rbIL-8, such as the extensive infiltration of PMNL and clot formation in mammary secretions, because one of the four cows given the high dose of rbIL-8 did not develop clinical mastitis or secrete elastase, LDP, or IL-8, and this was also true of the four cows given the low dose (10 *μ*g/quarter/head) of rbIL-8. Similarly, we previously showed that the intramammary infusion of rbIL-8 at 25 *μ*g, but not at 5 *μ*g, induced mastitic symptoms [25], suggesting that a threshold exists between 10 and 25 *μ*g of rbIL-8 per quarter that elicits significant local and/or systemic inflammatory reactions. In addition, these results suggest that individual differences in response to the intramammary infusion of rbIL-8 exist, as individual differences in the production of inflammatory cytokines in response to lipopolysaccharides are even observed in cultured dermal fibroblasts obtained from cows [37].

During the normal early dry-period, neutrophils are involved in tissue remodeling via the degradation of the basement membrane and extracellular matrix, and some of these cells infiltrate into mammary secretions [38, 39]. In this study, rbIL-8 infusion-induced mastitis caused a large number of PMNL to infiltrate into the mammary secretions. This recruitment of PMNL, especially neutrophils, is expected to induce further events. Neutrophils release both Lf [19] and proteases such as elastase [20, 21], resulting in the production of LDP [22]. Inflammatory LDP, especially peptides containing the GQRDLLFKDSAL sequence, such as the 22 and 23 kDa peptides, act on mammary epithelial cells to enhance IL-8 gene expression [22] and production. In the present study, the extensive infiltration of PMNL and high levels of elastase activity were found in the mammary secretions of the affected glands from days 1 to 28 PI. Moreover, the increased levels of the 22 and 23 kDa LDP and decreased Lf levels lasted for 28 days, which was suggestive of the sustained production of LDP by Lf cleavage. Furthermore, high levels of IL-8 were detected and sustained for 28 days PI. As the high levels of IL- 8 in the mammary secretions were unlikely to be explained by the ejection of the infused rbIL-8, we assumed that the 22 and 23 kDa LDP produced from Lf by elastase had induced the synthesis and secretion of IL-8. In addition, the long-lasting effects of the infusion such as the prolonged IL-8 secretion can be explained by assuming that the newly synthesized IL-8 repeatedly triggered new cycles of these sequential events. Although a major source of IL-8 secretion in bovine mastitic mammary glands was thought to be mammary epithelial cells [23, 24], leukocytes that have infiltrated the glands may be another source. To obtain better understanding of the roles of IL-8 in the pathogenesis of mastitis, cells that express IL-8 in the mastitic tissue should be examined.

In the normal bovine mammary involution process, especially at 3 to 14 days after dry-off, the mRNA expression and protein production of Lf are increased, as is its secretion into mammary secretions [40]. As elastase breaks down the Lf that is produced during mammary involution, in IL-8-induced mastitis, the Lf concentration in the mammary secretion might not recover during the 28 days after dry-off. It is reported that intact bovine Lf enhances the internalization of *Streptococcus uberis* and coagulase-negative staphylococci (CNS) into bovine mammary epithelial cells [41, 42], whereas a digestion product of Lf produced by proteases such as elastase, lactoferricin, which is derived from a different part of Lf from the 22 and 23 kDa LDP, has an antimicrobial effect [22]. Therefore, in the bovine mammary gland, IL-8 might be a key factor in the host defense system, contributing to the recruitment of neutrophils and the subsequent release of elastase, the digestion of intact Lf, and the production of LDP. 

Mastitis can be experimentally induced in dairy cows during lactation by the intramammary inoculation of *Escherichia coli*, *Mycoplasma bovis*, *Pseudomonas aeruginosa*, *S. aureus*, *S. epidermidis*, *S. simulans*, *Serratia marcescens*, or *Streptococcus uberis* [5–11]. In all cases, IL-8 is found in the milk, although the amount of IL-8 and the duration of its appearance depend on the pathogen used. Interestingly, while IL-8 disappears within a week in most cases, *S. aureus* inoculation results in a relatively long-lasting increase in IL-8 levels [6, 10]. *S. aureus* mastitis is one of the major types of bovine mastitis in the nonlactation and lactation periods [43]. This form of mastitis is often difficult to cure, especially in its chronic form [43–45]. As rbIL-8-induced mastitis displayed long-lasting symptoms, it is possible that the transition from the acute to chronic form of mastitis results in a lasting increase in IL-8 levels in *S. aureus* mastitis. In addition, in the present study, the mammary secretions from the infected glands of dry-period *S. aureus* mastitis-affected cows showed increased levels of PMNL, elastase, LDP, and IL-8; a decreased level of Lf; clot formation, a sign of *S. aureus* mastitis [46]. Therefore, the similarity between the results for rbIL-8- and *S. aureus*-induced mastitis suggest that the idea described above that IL-8 triggers repetitive cycles of sequential events leading to long-lasting inflammation might also apply to the development and progression of clinical dry-period *S. aureus* mastitis. 

In addition to *S. aureus* mastitis, prolonged IL-8 release into milk is observed in mastitis caused by CNS infection during lactation [11]. However, CNS infection-induced mastitis rarely causes severe clinical symptoms or milk clots, in contrast to *S. aureus* mastitis [47]. The differences in the clinical features of *S. aureus* and CNS mastitis indicate that prolonged IL-8 secretion in the mammary gland does not always lead to the same pathogenesis. Although IL-8 is undoubtedly involved in the pathogeneses of various types of mastitis [5–11, 48], other factors and conditions related to the causative pathogen that influence the pathogenesis of mastitis must be elucidated in future studies.

In summary, the present study demonstrated that a single intramammary infusion of rbIL-8 caused long-lasting PMNL infiltration and clot formation and the prolonged secretion of leukocyte elastase, inflammatory LDP, and IL-8 in dairy cows during the drying-off period. Similar changes were also observed in the secretions from mammary glands affected by clinical dry-period mastitis caused by *S. aureus* infection. These findings suggest the aforementioned assumption, but to obtain a better understanding of the role of IL-8 in the pathogenesis of *S. aureus* dry-period bovine mastitis, further studies in various phases of *S. aureus* mastitis and comparisons with CNS infection-induced mastitis are necessary.

## Figures and Tables

**Table 1:** Milk clot formation; PMNL count; elastase activity; concentrations of IL-8, 22 and 23 kDa LDP, and Lf in mammary secretions from the uninfected and infected quarters of cows with *S. aureus* dry-period mastitis.

	Quarter	
	Uninfected (*n* = 21)	Infected (*n* = 7)
*S. aureus* (log_10_ cfu/mL)	ND^1^	3.19 ± 0.17
Milk clot formation (number of quarters)	0	7
PMNL (log_10_ count/mL)	6.18 ± 0.04	> 8.0^2^
Elastase (units/mL)	ND	0.58 ± 0.11
IL-8 (pg/mL)	ND	1840 ± 581
22 and 23 kDa LDP (*μ*g/mL)	1.49 ± 0.39	35.7 ± 4.6^3^
Lf (mg/mL)	9.70 ± 0.23	3.53 ± 0.28^3^

^1^Not detectable.

^2^Due to the presence of milk clots, PMNL counts could not be precisely determined. PMNL counts of more than 10^8^ cells/mL were excluded.

^3^Significantly different compared to the uninfected quarters.
